# Methylphenidate during early consolidation affects long-term associative memory retrieval depending on baseline catecholamines

**DOI:** 10.1007/s00213-016-4502-8

**Published:** 2016-12-24

**Authors:** Isabella C. Wagner, Mariët van Buuren, Leonore Bovy, Richard G. Morris, Guillén Fernández

**Affiliations:** 1 Donders Institute for Brain, Cognition and Behaviour, Radboud University Nijmegen Medical Center, Kapittelweg 29, Nijmegen, 6525 EN The Netherlands; 2 Centre for Cognitive and Neural Systems, University of Edinburgh, Edinburgh, EH8 9JZ UK

**Keywords:** Ritalin, Dopamine, Norepinephrine, Synaptic tagging and capture, Systems consolidation, fMRI

## Abstract

**Rationale:**

Synaptic memory consolidation is thought to rely on catecholaminergic signaling. Eventually, it is followed by systems consolidation, which embeds memories in a neocortical network. Although this sequence was demonstrated in rodents, it is unclear how catecholamines affect memory consolidation in humans.

**Objectives:**

Here, we tested the effects of catecholaminergic modulation on synaptic and subsequent systems consolidation. We expected enhanced memory performance and increased neocortical engagement during delayed retrieval. Additionally, we tested if this effect was modulated by individual differences in a cognitive proxy measure of baseline catecholamine synthesis capacity.

**Methods:**

Fifty-three healthy males underwent a between-subjects, double-blind, placebo-controlled procedure across 2 days. On day 1, subjects studied and retrieved object-location associations and received 20 mg of methylphenidate or placebo. Drug intake was timed so that methylphenidate was expected to affect early consolidation but not encoding or retrieval. Memory was tested again while subjects were scanned three days later.

**Results:**

Methylphenidate did not facilitate memory performance, and there was no significant group difference in activation during delayed retrieval. However, memory representations differed between groups depending on baseline catecholamines. The placebo group showed increased activation in occipito-temporal regions but decreased connectivity with the hippocampus, associated with *lower* baseline catecholamine synthesis capacity. The methylphenidate group showed stronger activation in the postcentral gyrus, associated with *higher* baseline catecholamine synthesis capacity.

**Conclusions:**

Altogether, methylphenidate during early consolidation did not foster long-term memory performance, but it affected retrieval-related neural processes depending on individual levels of baseline catecholamines.

## Introduction

Memories for some experiences quickly fade while others persist for a lifetime. The process that converts and integrates initially fragile memories into a stable engram is referred to as memory consolidation (Dudai 2004; Squire et al. 2015). Foremost, consolidation involves changes at the synaptic level. According to the synaptic tagging and capture hypothesis (Frey and Morris 1997; and see Redondo and Morris 2011 for a reformulation and review), the encoding of new information triggers synaptic long-term potentiation (LTP) that results in neurochemical and structural alterations. Together, this creates the potential for a long-lasting synaptic change. The memory trace, however, is only stabilized and stored in long-term memory if these cellular events are accompanied by the synthesis of plasticity-related proteins (PRPs; Moncada and Viola 2007; Bekinschtein et al. 2008; Ballarini et al. 2009; Moncada et al. 2011). This cascade of events critically depends on the influx of catecholamines, such as dopamine (DA) and norepinephrine (NE), into the hippocampus (or other task-relevant brain regions; Ballarini et al. 2009). Blockade of the catecholaminergic transmitter system after encoding was shown to prevent long-term memory stabilization (Moncada and Viola 2007; Rossato et al. 2009; Moncada et al. 2011) but not immediate memory (Bethus et al. 2010). Facilitation of catecholamine signaling, on the other hand, was found to enhance memory persistence (Moncada and Viola 2007; Rossato et al. 2009; Wang et al. 2010; Moncada et al. 2011; McNamara et al. 2014). Evidence for the role of the synaptic tagging and capture hypothesis in long-term memory formation, however, mostly stems from evidence in rodents (but see Wetzel et al. 1981; Izquierdo et al. 2008). Therefore, we investigated if catecholamine modulation after encoding facilitated long-term memory stabilization in humans.

After initial synaptic modifications (mostly investigated) in the hippocampus, memories increasingly depend on neocortical structures as consolidation progresses (Marr 1970; Frankland and Bontempi 2005; Takashima et al. 2006; Takehara-Nishiuchi and McNaughton 2008; Lesburguères et al. 2011). Typically, successful retrieval of consolidated memories involves the medial prefrontal and posterior cingulate cortex, the angular gyrus, and posterior representational regions that code for specific features of the task material at hand (Rugg and Vilberg 2012; Wagner et al. 2015; King et al. 2015). Here, we hypothesized that catecholamine modulation would not only foster synaptic but also subsequent systems consolidation.

A stimulant that blocks both DA and NE reuptake (Volkow et al. 2001; Hannestad et al. 2010) and thereby increases catecholamine availability in the synaptic cleft is methylphenidate (MPH; Ritalin®). MPH is widely prescribed to alleviate symptoms of inattention and hyperactivity in attention-deficit/hyperactivity disorder (Faraone and Buitelaar 2010; Wigal et al. 2011) and is used by healthy individuals to improve academic performance (Greely et al. 2008; Smith and Farah 2011). Aside from its enhancing effects in multiple cognitive domains (see Linssen et al. 2014b for a review), few studies have investigated the effects of MPH on memory (Wetzel et al. 1981; Izquierdo et al. 2008; Linssen et al. 2012; Linssen et al. 2014a). For example, MPH given before encoding was found to facilitate the delayed but not the immediate recall of word lists (Linssen et al. 2012; Linssen et al. 2014a). However, MPH likely boosted attention at encoding, making it difficult to delineate its effects on post-encoding consolidation. Thus, we asked if catecholamine modulation by MPH after encoding would promote the synaptic and subsequent systems consolidation of associative memories.

**Fig. 1:**
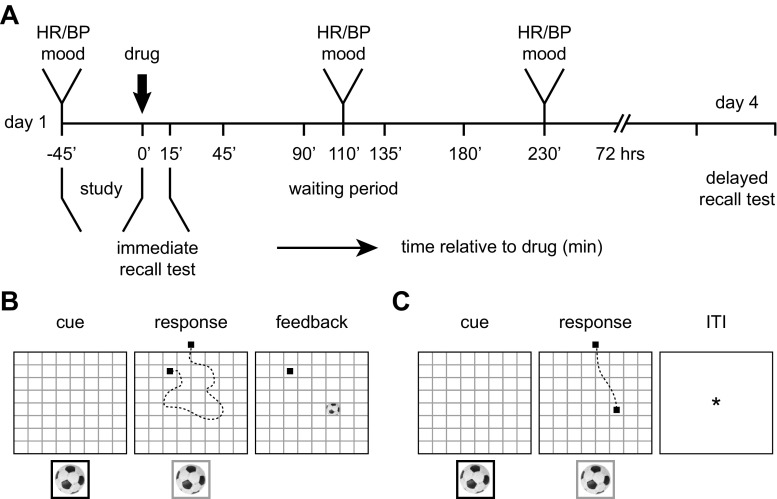
Study timeline and associative memory task. **a** Day 1 took place in the behavioral laboratory where subjects arrived at ∼10 a.m. They then studied and retrieved object-location associations and received 20 mg methylphenidate or placebo (*t* = 0). Peak drug effects were expected after the immediate recall test, during the waiting period (*t* = ∼110 min), where subjects were allowed to relax and watched nature documentaries. Heart rate (HR), blood pressure (BP), and mood (PANAS) were assessed at three times throughout day 1. On day 4, subjects were tested for all object-location associations inside the MR scanner (delayed recall test). **b** During study trials (behavioral lab, day 1), object-location associations were learned. First, subjects viewed the grid with all objects placed at their correct locations for 1.5 min (not depicted in figure). This was followed by five study cycles, each containing study trials for all object-location associations. A trial started with the presentation of an empty grid and the object, surrounded by a red frame (here in *black*), at the bottom of the screen (cue, 3 s). As the frame turned green (here in *grey*), a cursor (marked as *black square*) appeared randomly at one of the four sides of the grid and the subject was required to indicate the correct object-location association (response, 2 s; exemplary cursor trajectory is marked as *dashed line*). If the correct object-location was selected, the object was shown in that location. If an incorrect object-location was selected (as illustrated), the cursor turned red (here *black*) and the object was shown in its correct location (feedback, 3 s). **c** During the immediate, as well as the delayed recall test, subjects were tested for their memory of all object-location associations. The trial started with the presentation of the empty grid and the object, surrounded by a black frame, at the bottom of the screen (cue, 3 s). As the frame turned green (here in *grey*), the cursor appeared randomly at one of the four sides of the grid, and the subject was required, indicating the correct object-location association (response, 2.5 s). The figure shows an example of a correct answer, and the cursor trajectory is marked as a *dashed line*. After responding, the cursor turned grey for the remaining response period (not shown), and no feedback was provided. The next trial started after a variable inter-trial interval (ITI; mean = 5 s, range = 2.5–7 s)

The present study spanned across 2 days that were 72 h apart (Fig. 1a). After studying object-location associations on day 1 (comparable to van Buuren et al. 2014), subjects received a single, oral dose of either MPH or placebo and were tested immediately for their initial memory (immediate recall test). Based on the pharmacological profile of MPH (Swanson and Volkow 2003), we expected a drug effect on brain function after the immediate recall test, during early consolidation, affecting memory 3 days later. Up-regulation of catecholaminergic signaling should then foster LTP and PRP release and promote synaptic and subsequent systems consolidation (Frey and Morris 1997; Frankland and Bontempi 2005). Long-term associative memory was tested again after about 72 h (day 4) during the delayed recall test while subjects were scanned using functional MRI.

We expected that MPH during early consolidation would stabilize initially fragile memories that would otherwise decay and enhance long-term associative memory performance in the MPH compared to the placebo group. This should be paralleled by increased engagement of neocortical regions during correct memory retrieval in MPH relative to placebo subjects, including medial prefrontal and posterior cingulate regions, the angular gyrus, and posterior representational areas. Additionally, the effects of catecholaminergic drugs on cognitive performance were previously shown to depend on individual working memory capacity which serves as a proxy for baseline catecholamine synthesis capacity (Cools et al. 2008; Landau et al. 2009). While task performance in subjects with low working memory capacity typically benefits from the administration of catecholaminergic stimulants (Mehta et al. 2000; Gibbs and D’Esposito 2005), it can have detrimental effects in subjects with high working memory capacity (Kimberg et al. 1997). Thus, we stratified our behavioral and neuronal effects with working memory capacity to take into account individual differences in baseline catecholamine synthesis capacity.

## Materials and methods

### Subjects

Sixty healthy males (mean = 23 years, age range = 18–31 years) volunteered for this study and provided written informed consent prior to participation. A total of seven subjects were excluded from the study: three subjects did not return after the intake procedure (Materials and methods, Intake procedure), one subject did not perform the task as instructed, two subjects were excluded due to a mistake by one of the experimenters, and one subject was excluded due to an incidentally found brain abnormality. This left 53 subjects (mean = 23 years, age range = 18–31 years) for the final analysis. The experimental protocol was reviewed and approved by the institutional review board (CMO Region Arnhem-Nijmegen, The Netherlands; registration number 2014/289). Recruitment took place via the subject database of the Radboud University (radboud.sona-systems.com) and flyers. Subjects received monetary compensation for participation.

### Intake procedure

All subjects underwent an intake procedure in the form of a personal interview. This consisted of a medical screening to determine whether the subject met all of the inclusion and none of the exclusion criteria. Blood pressure and heart rate were measured using a digital blood pressure monitor (Omron Healthcare Europe B.V., The Netherlands). Inclusion criteria were as follows: age between 18 and 35 years, normal or corrected-to-normal vision, no current disease, male, and right-handed. Exclusion criteria were as follows: (history of) psychiatric, neurological, or endocrine treatment; autonomic failure (e.g., vasovagal reflex syncope); (history of) clinically significant hepatic, cardiac, obstructive respiratory, renal, cerebrovascular, metabolic, or pulmonary disease; family history of sudden death or ventricular arrhythmia; (history of) epilepsy; (history of) drug (opiates, LSD, (meth-) amphetamines, cocaine, solvents, barbiturates) or alcohol dependence; family history of schizophrenia or bipolar disorder; current or past use of psychotropic medication; regular use of corticosteroids; suicidality; diabetes; uncontrolled hypertension (defined as diastolic blood pressure at rest >95 mmHg or systolic blood pressure at rest >180 mmHg); hypotension (defined as diastolic blood pressure <50 mmHg or systolic blood pressure <95 mmHg, or resting pulse rate <45 beats/min); abnormal hearing or (uncorrected) vision; lactose intolerance; irregular sleep/wake rhythm (e.g., regular nightshifts or cross-timeline travel); current use of oral medication aside from occasional use of Paracetamol®; any personal characteristics that make the subjects ineligible to enter the MR scanner such as non-removable metallic objects in the body, active implants (e.g., pacemaker, neurostimulator), claustrophobia, head surgery, or metallic tattoos.

The intake procedure lasted ∼30 min. The final checklist was signed off by one of the experimenters (authors ICW, MvB, or LB) and the responsible study physician (last author GF). If the subject was eligible for study participation, day 1 was scheduled within 4 weeks after the intake procedure. This was not possible for three subjects. For those, another intake procedure was scheduled before the start of day 1.

### Study procedure

The study consisted of a between-subjects, double-blind, placebo-controlled procedure across 2 days. Day 1 took place in the behavioral laboratory where subjects studied and retrieved 64 object-location associations (Materials and methods, Associative memory task) and received either 20 mg MPH or placebo. They were again tested for all object-location associations during a delayed recall test inside the MR scanner about 72 h later (day 4; Fig. 1a).

Subjects arrived at ∼10 a.m. on day 1. They were tested in the behavioral laboratory in groups of maximum three, but seated in separated cubicles so that interactions were minimized. First, we asked if subjects had refrained from alcohol, other drugs, and medication within the 24 h prior to the start of day 1, and if they had undergone a medical examination since the intake procedure. Next, we asked if subjects had consumed caffeinated drinks or had smoked in the morning. No subject had to be excluded because of these restrictions. Furthermore, subjects were instructed to have a light breakfast 1 h before arrival. If they had not done so, they were offered a small snack consisting of breakfast cookies and water. Subjects subsequently completed a set of questionnaires, including the Behavioural Inhibition Scale/Behavioural Activation Scale (BIS/BAS; Carver and White 1994), the Barratt Impulsiveness Scale (Patton et al. 1995), and the ADHD Rating Scale-IV (DuPaul et al. 1998) to assess baseline levels of impulsivity and ADHD symptoms, respectively. The Positive and Negative Affect Scale (PANAS; Watson et al. 1988) was used to assess the current mood (*t* = −45 min before drug intake). Next, heart rate and blood pressure were measured, and subjects were instructed for the associative memory task and performed the study phase of the task (Materials and methods, Associative memory task).

After the study phase and before the immediate recall test of the associative memory task, subjects received one oral capsule of 20 mg MPH or placebo in a double-blind, randomized fashion (*t* = 0 min; Materials and methods, Study medication, randomization, and un-blinding procedure). Following intake, they then performed the immediate recall test (Materials and methods, Associative memory task). Plasma levels of MPH peak 1.5–2 h (*t*_max_) after drug intake (Swanson and Volkow 2003) and we therefore reasoned that the *t*_max_ would be reached after the immediate recall test.

The pharmacological effects of MPH diminish with a half-life of 2–3 h (Swanson and Volkow 2003). To control activity during the consolidation window (i.e., no arousing activity that could trigger the additional release of catecholamines) and to secure the subjects’ well-being following drug intake, subjects remained in the behavioral laboratory for ∼3.5 h after completing the immediate recall test. During this period, they were allowed to relax and watch nature documentaries from a desk chair (Planet Earth, Life, BBC, 2009). Blood pressure, heart rate, and mood measures (PANAS) were obtained *t* = 110 min after drug intake (i.e., ∼90 min after the immediate recall test), as well as *t* = 230 min after drug intake (i.e., ∼3.5 h after the immediate recall test), and subjects were allowed to consume snacks (sandwiches, cookies, water) after the *t* = 110 min measurement. In total, the session on day 1 took ∼5 h. The responsible study physician (last author GF) was on call within the building at all times during day 1.

After 72 h, subjects returned to the laboratory and were placed in the MR scanner (day 4; day 4–day 1 difference: mean = 72 h; range = 70–73 h). They received the task instructions and underwent a brief resting-state scan (11 min), a structural scan during which they could practice using the MR-compatible trackball (5 min), and the delayed recall test of the associative memory task (Materials and methods, Associative memory task). Lastly, we assessed the working memory capacity outside the scanner (Materials and methods, Working memory capacity), subjects were debriefed about the purpose of the study, and were asked if they thought that they had received MPH or placebo (“Do you think you received methylphenidate or placebo? How sure are you (0–100)?”). The session on day 4 took ∼1.5 h.

### Associative memory task

The associative memory task consisted of a study phase (day 1) and two memory tests (immediate recall test, day 1; delayed recall test inside the MR scanner, day 4; Fig. 1a). On day 1, subjects were instructed to memorize the locations of 64 different objects (Hemera Photo-Object database, Hemera Technologies) that were placed on an 8 × 8 grid (see also van Buuren et al. 2014). A trackball (Orbit Optical Trackball, Kensington) was used to perform the task and subjects first completed a short practice round for familiarization. Following this, the study phase of the associative memory task started and subjects viewed the grid with all objects placed at their correct locations for 1.5 min. They then completed five study cycles that each contained 64 trials to study all object-location associations. A trial started with the presentation of the empty grid and an object presented at the bottom of the screen, surrounded by a red frame (3 s; Fig. 1b). As soon as the frame turned green, a cursor appeared randomly at one of the four sides of the grid (this random start position was implemented to avoid motor preparation), and the correct object-location had to be indicated by scrolling to and clicking on the respective location on the grid (2 s). After responding, feedback was provided on screen for 3 s plus the remaining response period. If the incorrect location was selected, the frame turned red and the object was displayed at the correct location. If the correct card was selected, the object was shown at that location. After each learning cycle, subjects received a short break of 30 s, indicated by a fixation cross presented on the computer screen and the next study cycle started. The study phase took ∼45 min. Object-location pairings were randomized across subjects and the presentation order was randomized within study cycles.

The immediate recall test (day 1) took place shortly after the study phase, immediately following the drug intake (Materials and methods, Procedure). Subjects were tested for their memory of all 64 object-location associations. Again, a trial started with the presentation of the empty grid and an object placed at the bottom of the computer screen, surrounded by a black frame (3 s; Fig. 1c). As the frame turned green, a cursor appeared randomly at one of the four sides of the grid and subjects had to indicate the correct location of the object by scrolling to and clicking on the grid location (2.5 s). After responding, no feedback was provided, but the cursor turned grey for the remaining response time and the next trial started after a variable delay of 2.5–7.5 s (mean = 5 s) during which an asterisk was presented on the screen. A short break of 30 s was given every 16 trials. Trial presentation was randomized across subjects and the immediate test lasted ∼15 min.

The delayed recall test (day 4, inside the MR scanner) was identical to the immediate recall test (day 1), only the order of the trials was again randomized. An identical trackball was in-house adapted for MR compatibility and placed on the subjects’ belly during scanning. Subjects again received a short practice before the beginning of the task. The entire associative memory task was programmed and presented with presentation (Version 16.4, www.neurobs.com).

### Working memory capacity

Previously, baseline catecholamine synthesis capacity was shown to correlate with individual working memory capacity (Cools et al. 2008; Landau et al. 2009) which is regarded as a stable, trait-like measure (Ilkowska and Engle 2010; Engle 2010). We assessed working memory capacity using the Dutch version of the Listening Span Task (Daneman and Carpenter 1980). In this task, subjects listened to sets of 1–7 sentences (that is, 3 sets per working memory level 1–7). For each sentence, a written factual verification question had to be answered. After the last sentence of each set, subjects were asked to turn the page and retrieve the final words of each sentence in the order in which they were presented. The listening span, or working memory capacity, represents the maximum number of sentences that were answered correctly (i.e., correct factual verification *and* correctly retrieved final word for all sentences in a set) on at least 2 out of 3 sets per working memory level. The maximum score was 7. If 1 set (out of 3) was answered correctly on the next highest working memory level, then this was scored with 0.5 points extra. The task was completed at the end of day 4 (∼25 min, outside the MR scanner) since we wanted to avoid any interfering effects of other tasks on associative memory encoding and consolidation during day 1.

### Study medication, randomization, and un-blinding procedure

Subjects orally received one capsule of either 20 mg MPH (Ritalin®) or placebo (lactose product) after the study phase of the associative memory task (Materials and methods, Study procedure). The assignment to MPH and placebo groups was randomized in groups of 10 subjects and the subjects, as well as the research team were blind to the randomization. Study medication and randomization list were prepared by the Department of Clinical Pharmacy (Radboud University Medical Center, Nijmegen, The Netherlands). After inclusion of all subjects, drug conditions were coded with *A* and *B* by an independent researcher and the un-blinding was done after the analysis of the critical outcome measures was finished (i.e., analysis of physiological and behavioral data).

### Physiological and behavioral data analysis

Heart rate, blood pressure, and mood (PANAS) were measured 3 times throughout day 1 (Fig. 1a). These measures were each analyzed using a mixed ANOVA with time (1–3) as a within-, and drug (MPH, placebo) as a between-subjects factor. Memory performance was defined as the number of correct responses in the associative memory task (Materials and methods, Associative memory task) for the study cycles (1–5, day 1), the immediate recall test (day 1), and for the delayed recall test (day 4). Performance and corresponding reaction times (RTs) for correct responses from the study phase were analyzed using mixed ANOVAs with Study Cycle (1–5) as a within-, and Drug (MPH, placebo) as a between-subjects factor. Memory performance for correct responses from the recall tests were analyzed using a time (immediate, delayed) × drug (MPH, placebo) mixed ANOVA. For the delayed recall test, only object-location associations that were retrieved correctly at both the immediate and the delayed recall test were regarded as “correct” (see also fMRI analyses below). Corresponding RTs were analyzed using two independent-samples *t* tests, since immediate and delayed recall tests were performed in the behavioral laboratory and inside the MR scanner, respectively, and are thus not directly comparable. Subjects displayed very few responses that were incorrect at the immediate but correct at the delayed recall test (“forgotten-remember responses”; mean ± SEM: 2.9 ± 0.25 trials) and few trials without any responses (“misses”; study, 2.9 ± 0.5 trials; immediate recall test, 1.4 ± 0.2 trials; delayed recall test, 3.5 ± 0.4 trials). These trials were excluded from the behavioral analysis, and were collapsed in a regressor of no interest for fMRI analyses of the delayed recall test on day 4 (see below). For all ANOVAs, we applied Greenhouse-Geisser correction whenever the assumption of sphericity was violated and significant interactions were followed up using independent-samples *t* tests. Alpha was set to 0.05 throughout. Additionally, we expected the effects of MPH administration on physiological and behavioral outcome measures to be affected by individual working memory capacity. We therefore repeated the above analyses with working memory capacity (mean centered) as a covariate.

### Imaging parameters

Imaging data were acquired using a 3 Tesla MRI scanner (Skyra, Siemens, Erlangen, Germany) using a 32-channel head coil. We obtained 364 T_2_*-weighted BOLD images during the delayed recall test (day 4). Parameters were as follows: gradient multi-echo EPI sequence (Poser et al. 2006), TR = 2100 ms, TEs = 8.5, 19.3, 30, 41 ms, flip angle = 90°, FOV = 224 × 224 mm, matrix = 64 × 64, 34 ascending axial slices, 17% slice gap, voxel size = 3 mm. Structural scans were acquired using a Magnetization-Prepared Rapid Gradient Echo (MP-RAGE) sequence with the following parameters: TR = 2300 ms, TE = 3.03 ms, flip angle = 8°, FOV = 256 × 256 mm, voxel size = 1 mm isotropic.

### MRI data preprocessing

All imaging data were analyzed using SPM8 (http://www.fil.ion.ucl.ac.uk/spm/) in combination with MATLAB (MATLAB 2014, The Mathworks, Inc., Natick, MA, USA). First, echoes from the four different echo times were combined into single volumes. We used 32 scans that were acquired before the start of the delayed recall test (∼1 min) to determine the optimal weighting of echo times for each voxel. This was done by calculating the contrast-to-noise ratio for each echo per scan. Images from multiple echo times were then combined by performing motion correction on the first echo, estimating iterative rigid body realignment to minimize the residual sum of squares between the first echo of the first scan and all remaining scans. The estimated parameters were then applied to all other echoes, realigning all echoes to the first echo of the first scan. Finally, the calculated optimal echo time weightings were used to combine the four echo images into a single image. The structural scan was co-registered to the mean functional scan. Next, using unified segmentation (Ashburner and Friston 2005), the structural scan was segmented and the normalization parameters were estimated using these normalization parameters. All images (functional and structural) were spatially normalized to the Montreal Neurological Institute (MNI) EPI template and smoothed with a 3D Gaussian kernel (8 mm full-width at half maximum, FWHM).

### fMRI activation analysis

To investigate differential activation of MPH and placebo groups during memory retrieval (delayed recall test, day 4), we sorted trials based on individual memory performance and grouped them in three regressors: First, correct trials were defined as object-location associations that were correctly remembered at both the immediate recall test (day 1) and the delayed recall test (day 4). Second, incorrect trials were defined as object-location associations that were incorrectly remembered at the delayed recall test (day 4). Third, object-location associations that were correctly remembered at the delayed recall test (day 4) but incorrectly remembered at the immediate recall test (day 1) were collapsed together with misses (trials without any response) into a regressor of no interest. The blood oxygen level-dependent (BOLD) response for all trials was modeled with these separate task regressors, time-locked to the onset of the trials. All events were estimated as a boxcar function with the duration of one trial (5.5 s) and convolved with a canonical hemodynamic response function. Regressors for correct and incorrect responses were parametrically modulated with RTs. In addition, the six realignment parameters were included in the design matrix, and a high-pass filter with a cut-off at 128 s was applied. General activation during the delayed test was assessed with a one-sample *t* test of the contrast correct > incorrect. Retrieval-related brain activity was compared between groups using an independent-samples *t* test (correct > incorrect). Unless stated otherwise, activation was tested for significance using cluster inference with a cluster-defining threshold of *P* < 0.005 and a cluster probability of *P* < 0.05 family-wise error (FWE) corrected for multiple comparisons. Furthermore, we expected that the effects of MPH administration on retrieval-related brain activity would depend on individual differences in working memory capacity. This was investigated with an independent-samples *t* test (correct > incorrect) and working memory capacity was added as a covariate of interest.

### fMRI connectivity analysis

As an exploratory step, we investigated connectivity during memory retrieval (delayed recall test, day 4) and its association with working memory capacity using psychophysiological interaction analysis (PPI; Friston et al. 1997). Seed regions were defined based on analyses of the association between retrieval-related activation (correct > incorrect) and working memory capacity, separately for each group (two one-sample *t* tests; Results, Activation and connectivity during memory retrieval 72 h after drug intake depend on working memory capacity; see also Table 2). For the placebo group, we placed a seed in the left lateral occipital cortex (*x* = −52, *y* = −63, *z* = 0; 8 mm sphere around peak coordinate). For the MPH group, a seed in the right postcentral gyrus was used (*x* = 66, *y* = −14, *z* = 18; 8 mm sphere around peak coordinate). For each seed, the first eigenvector of the time course was extracted (i.e., the physiological factor) and adjusted for average activation during the task (*F*-contrast). This time course was then convolved with the respective task condition (i.e., the psychological factor; contrast correct > incorrect) and increased connectivity with the seed region during correct > incorrect retrieval was investigated. The resulting individual PPI contrast images for each seed region were submitted to one-sample *t* tests per drug condition (MPH, placebo), and working memory capacity was added as a covariate of interest (see above). Again, unless stated otherwise, significance was tested using cluster inference with a cluster-defining threshold of *P* < 0.005 and a cluster probability of *P* < 0.05 FWE-corrected for multiple comparisons.

### Scan-to-scan motion between groups

Finally, we assured that our fMRI results were not confounded by unequal magnitude of scan-to-scan motion between the two groups during memory recall. We calculated the framewise displacement (*FD*) for every scan at time *t* by *FD*(*t*) = |Δ*dx*(*t*)| + |Δ*dy*(*t*)| + |Δ*dz*(*t*)| + *r*|*α*(*t*)| + *r*|*β*(*t*)| + *r*|*γ*(*t*)|, where (*dx*, *dy*, *dz*) is the translational and (*α*, *β*, *γ*) the rotational movement (Power et al. 2012). The average *FD* during memory recall was generally small (mean ± SEM, MPH: 0.13 ± 0.01 mm; placebo: 0.13 ± 0.01 mm) and did not significantly differ between groups (*P* = 0.703). Thus, the amount of movement during the delayed recall was comparable between subjects who had received MPH or placebo.

## Results

### Subject sample

From our final sample of 53 subjects, 26 subjects received 20 mg MPH (mean = 24 years, age range = 18–31 years) and 27 subjects received placebo (mean = 23 years, age range = 19–29 years) at *t* = 0 min (Fig. 1a). Within the MPH group, 14 out of 26 subjects (54%) correctly guessed that they had received MPH with a certainty of 66%. Twenty-four out of 27 subjects (89%) correctly guessed that they had received placebo with a certainty of 73%.

Several self-report questionnaires were completed at baseline on day 1 (Materials and methods, Study procedure). The groups did not significantly differ in behavioral inhibition (BIS; mean ± SEM, MPH: 17.8 ± 3, placebo: 17.7 ± 3.4, *P* = 0.908), but the MPH group reported increased behavioral activation (BAS; MPH: 25 ± 4.1, placebo: 22.9 ± 3.2, *P* = 0.045; not significant using a Bonferroni alpha of 0.008). There was no significant difference in trait impulsivity between groups (Barratt Impulsiveness Scale; MPH: 63 ± 9.5, placebo: 65.6 ± 9.6, *P* = 0.330). Further, there was no significant group difference in ADHD symptoms of inattention (ADHD Rating Scale-IV; MPH: 19 ± 4.3, placebo: 20 ± 5.6, *P* = 0.489) or impulsivity (MPH: 4.5 ± 1.2, placebo: 5.2 ± 1.6, *P* = 0.086), but placebo subjects reported more symptoms of hyperactivity (MPH: 15 ± 3.1, placebo: 16.8 ± 3, *P* = 0.043; not significant using a Bonferroni alpha of 0.008). However, there was no reliable relation between any of the above measures with associative memory performance on day 4 (Results, Associative memory performance; all *P* > 0.05). Further, MPH and placebo groups did not differ significantly in terms of working memory capacity (Listening Span Task; MPH: 4.7 ± 1.2, placebo: 5.2 ± 0.9, *P* = 0.102).

### Physiological and psychological effects of drug administration

**Fig. 2:**
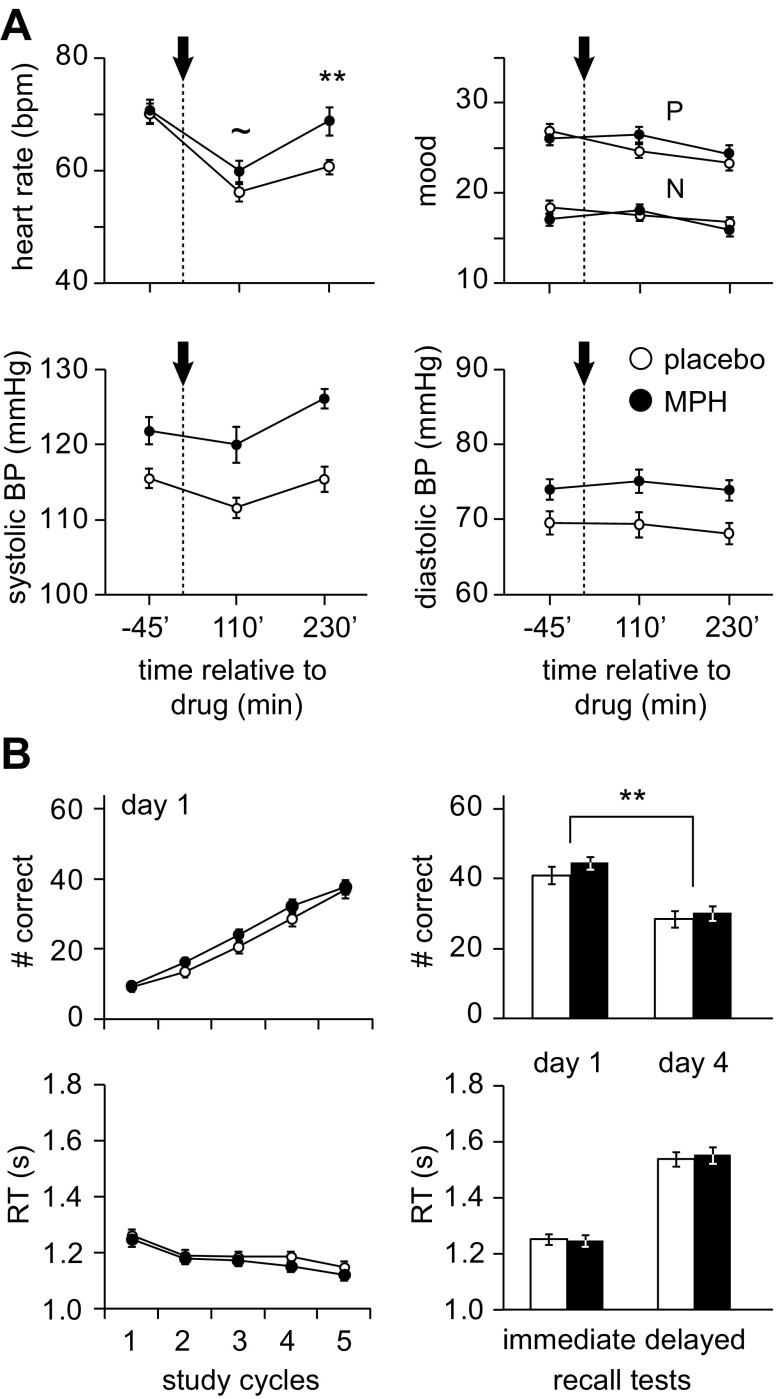
Effects of drug intake on physiological, mood, and associative memory measures. **a** Heart rate (beats/min, bpm), mood (P, positive; N, negative), and blood pressure (BP, mmHg) were assesses three times on day 1. *t* = 0 marks the drug intake, indicated through *dashed line* and *arrow*. **b** *Upper panel*: associative memory performance (number of correct object-location associations) throughout study cycles on day 1 and the immediate (day 1), as well as the delayed recall test (day 4). *Lower panel*: reaction times (s) for correct responses during the associative memory task. All *error bars* denote ± standard error of the mean (SEM). *White circles*/*bars* indicate placebo group, *black circles/bars* indicate methylphenidate group (MPH). ** denotes *P* < 0.001, ∼ trend-level significance

To start out, we investigated the effects of drug administration on heart rate, blood pressure, and mood (PANAS) on day 1. We found a decrease in heart rate across day 1 (main effect of time: *F*(1.8,91.4) = 69, *P* < 0.0005) and a time × drug interaction (*F*(1.8,91.4) = 6.3, *P* = 0.004; no main effect of drug: *P* = 0.058). This was caused by an increase (or less of a decrease) in heart rate of the MPH group at *t* = 230 min after drug intake (*t*(37.5) = −2.9, *P* = 0.005). There was no significant difference in heart rate between MPH and placebo groups at *t* = −45 min before drug intake (*P* = 0.825) and a trend-level increase (or less of a decrease) at *t* = 110 min after drug intake for subjects who received MPH (*t*(47.3) = −1.9, *P* = 0.066; Fig. 2a, left upper panel).

Systolic blood pressure was higher in the MPH group (main effect of drug: *F*(1,51) = 20.5, *P* < 0.0005) but did not depend on or significantly interact with the drug manipulation (no main effect of time: *P* = 0.077; no time × drug interaction: *P* = 0.721). Similarly, we found higher diastolic blood pressure for the MPH group (main effect of drug: *F*(1,51) = 7.5, *P* = 0.008), but again unrelated to the drug manipulation (no main effect of time: *P* = 0.282; no time × drug interaction: *P* = 0.471; Fig. 2a, lower panels).

Positive mood did not differ systematically between the two groups but showed a general decrease over the course of day 1 (main effect of time: *F*(1.7,84.5) = 15, *P* < 0.0005, no main effect of drug: *P* = 0.541; interaction time × drug: *F*(1.7,84.5) = 4, *P* = 0.030), as did negative mood (main effect of time: *F*(2,102) = 10.6, *P* < 0.0005, no main effect of drug: *P* = 0.532; interaction time × drug: *F*(2,102) = 3.3, *P* = 0.042; post hoc comparisons at *t* = −45 min, *P* = 0.199; at *t* = 110 min, *P* = 0.633; at *t* = 230 min, *P* = 0.367; Fig. 2a, right upper panel). Results for heart rate, blood pressure, and mood did not change when we repeated the analyses with working memory capacity as a covariate.

In sum, the MPH group (compared to the placebo group) exhibited less of a decrease in heart rate after drug intake on day 1. Blood pressure was generally higher in the MPH group. Positive and negative mood ratings decreased throughout day 1 in both groups. Both, blood pressure and mood, were unaffected by the drug manipulation.

### Drug effects on associative memory performance

Associative memory performance was defined as the number of correctly remembered object-location associations. Subjects showed an increase in performance over the study cycles on day 1 (main effect of time: *F*(1.8, 91.6) = 352.3, *P* < 0.0005; Fig. 2b, left upper panel). As expected, this increase did not differ significantly between groups (no main effect of drug: *P* = 0.304; no time × drug interaction: *P* = 0.201), since the drug was administered after the completion of the study phase (Materials and methods, Procedure; Fig. 1a). Also, RTs for correct responses did not significantly differ between the two groups and showed a general decrease across study cycles (main effect of time: *F*(2.8,137.6) = 20.5, *P* < 0.0005; no main effect of drug: *P* = 0.461; no time × drug interaction: *P* = 0.831; Fig. 2b, left lower panel).

After study, subjects received an oral dose of either 20 mg MPH or placebo and subsequently performed the immediate recall test (day 1). Seventy-two hours later, subjects performed the delayed recall test (Materials and methods, Procedure). Memory performance decreased over the course of 72 h (main effect of time: *F*(1,51) = 374.9, *P* < 0.0005), but this decrease did not significantly differ between the MPH and placebo groups (no main effect of drug: *P* = 0.389; no time × drug interaction: *P* = 0.212; Fig. 2b, right upper panel). Comparably, RTs for correct responses at the immediate and delayed recall tests did not significantly differ between MPH and placebo groups (immediate recall test: *P* = 0.874; delayed recall test: *P* = 0.727; Fig. 2b, right lower panel). Results remained stable when we re-analyzed associative memory performance and RTs with individual working memory capacity as a covariate.

In conclusion, both groups showed a steady increase in associative memory performance throughout the study cycles on day 1, parallelized by a decrease in RTs for correct responses. Also, performance at the immediate recall test (day 1) was high and did not differ between groups. Contrary to what we expected, the MPH group did not show better memory performance after 72 h.

### Activation during memory retrieval 72 h after drug intake

**Fig. 3:**
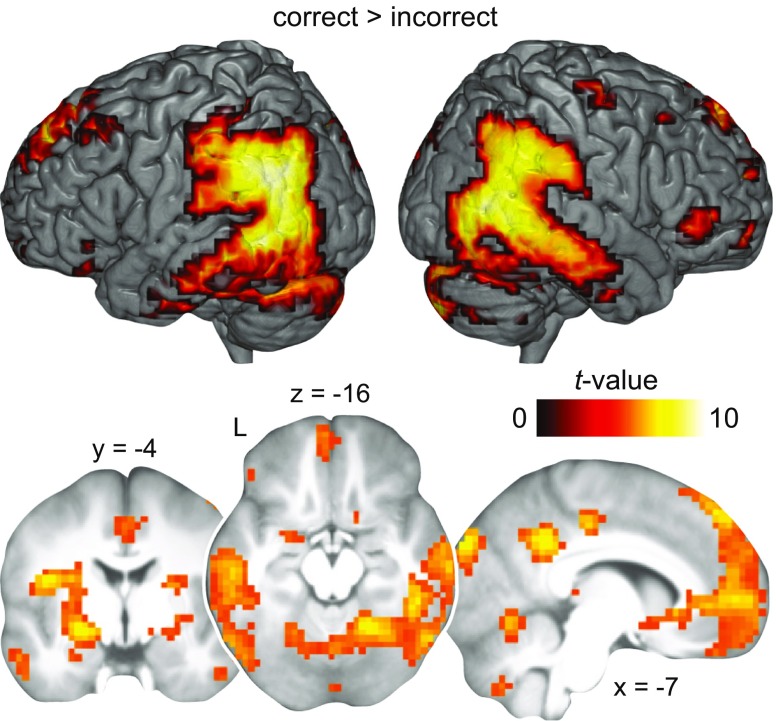
Activation during memory retrieval 72 h after drug intake. Increased BOLD activation across all subjects (both groups; *N* = 53) during memory retrieval of correct compared to incorrect object-location associations on day 4. Results are shown at *P* < 0.05 FWE-corrected (Table 1). Sliced images are based on the average structural scan of all subjects. *L* left

To verify that correct memory retrieval was associated with reliable, neocortical engagement on day 4, we first investigated activation collapsed across both groups. Overall, we found increased activation in the hippocampus and surrounding MTL structures, medial prefrontal and posterior cingulate cortex, angular gyrus, striatum, middle and inferior temporal structures, including the fusiform gyrus and the lateral occipital cortex during correct > incorrect memory retrieval (Fig. 3, Table 1). Next, we hypothesized that the MPH compared to the placebo group would show stronger engagement of neocortical regions during correct memory retrieval on day 4, including the medial prefrontal and posterior cingulate cortex, as well as the angular gyrus and posterior representational areas. However, we did not find differential activation between the MPH and placebo group, also not at a more liberal threshold (*P* < 0.005, uncorrected; contrasts MPH > placebo, placebo > MPH). Thus, correct (compared to incorrect) retrieval yielded stronger activation in the hippocampus and neocortical regions that did not significantly differ between groups.

**Table 1:** Activation during memory retrieval 72 h after drug intake

		MNI			
Contrast and brain regions	*x*	*y*	*z*	*Z* value	Cluster size
All subjects, *N* = 53 Correct > incorrect					
L middle temporal gyrus	−46	−52	4		4692
L cuneus	0	−88	24	6.76	462
L superior frontal gyrus	−14	46	46	6.58	1693
R cingulate gyrus	4	−21	42	6.37	182
R inferior frontal gyrus	49	38	4	6.27	44
R precentral gyrus	42	−10	63	5.79	24
R middle frontal gyrus	32	63	−7	5.66	19
L middle frontal gyrus	−38	24	46	5.56	33
L inferior frontal gyrus	−32	49	−10	5.41	9
R inferior temporal gyrus	49	−7	−35	5.37	14

MNI coordinates represent the location of peak voxels. We report the first local maximum within each cluster. Effects were thresholded at *P* < 0.05, FWE-corrected for multiple comparisons. Anatomical nomenclature was obtained from the Laboratory for Neuro Imaging (LONI) Brain Atlas (LPBA40; http://www.loni.usc.edu/atlases/)

*L* left, *R* right

### Activation and connectivity during memory retrieval 72 h after drug intake depends on working memory capacity

**Fig. 4:**
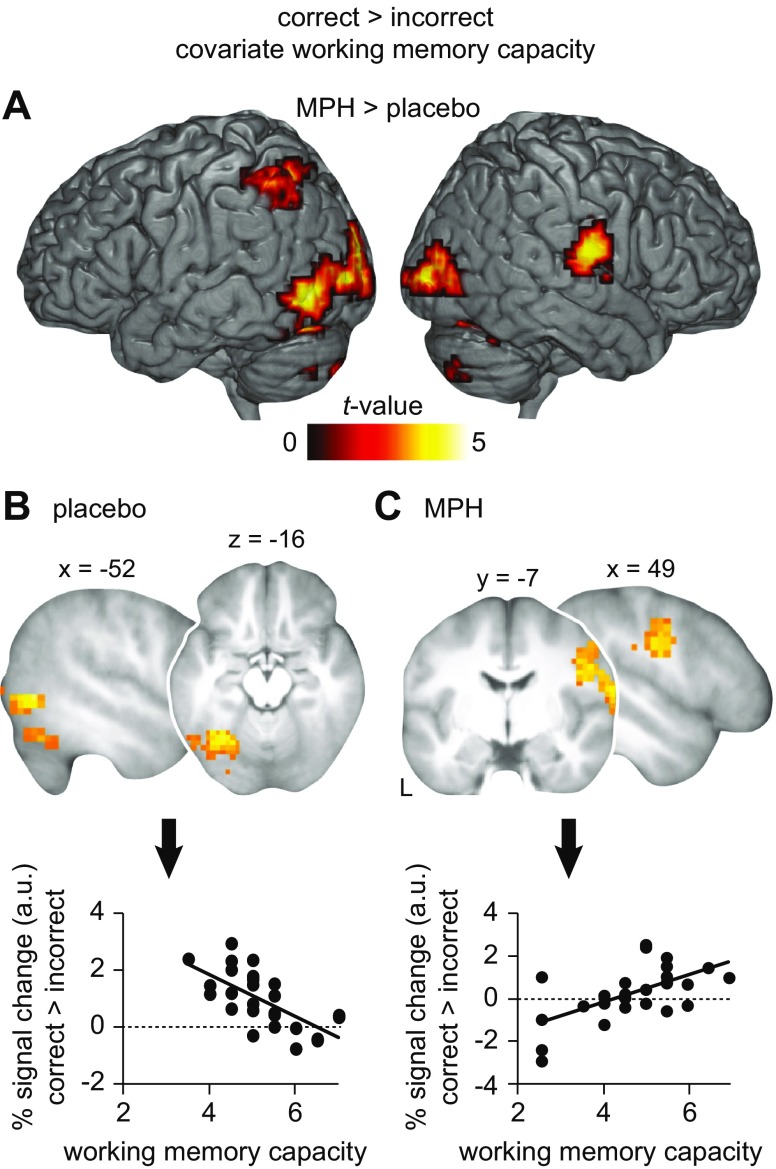
Activation during memory retrieval 72 h after drug intake depends on working memory capacity. **a** The relationship between correct > incorrect retrieval-related BOLD activation and individual working memory capacity is different for the methylphenidate (MPH) and placebo groups. **b**–**c** *Upper panels*: the significant effects from **a** were followed up with one-sample *t* tests for each group, for the contrast correct > incorrect, and again with working memory capacity added as a covariate of interest. **b** Increased activation during memory retrieval in placebo subjects (*N* = 27) is related to lower working memory capacity. **c** Increased activation during memory retrieval in MPH subjects (*N* = 26) is related to higher working memory capacity. For visualization purposes, *left* and *right* *lower panels* show the relationship between working memory capacity and the % signal change during correct > incorrect retrieval (arbitrary units, a.u.), extracted from the significant clusters. Results are shown at *P* < 0.005 (*P* < 0.05, FWE-corrected at cluster level; see also Table 2). Sliced images are based on the average structural scan of all subjects. *L* left

Lastly, we hypothesized that the effects of drug administration on retrieval-related activation would linearly depend on individual variation in working memory capacity, a proxy measure for baseline catecholamine synthesis capacity. We found that working memory capacity was associated with increased activation in the left lateral occipital cortex, fusiform gyrus, left angular gyrus, and the right postcentral gyrus during correct relative to incorrect retrieval in MPH compared to placebo subjects (MPH > placebo, no difference for placebo > MPH; correct > incorrect; Fig. 4a, Table 2). Hence, the linear relationship between retrieval-related activation and individual working memory capacity differed between groups. To follow-up on this, we tested the association between individual working memory capacity and retrieval-related activation separately for each group using one-sample *t* tests, again with working memory capacity added as a covariate of interest. In the placebo group, increased activation in left fusiform gyrus and lateral occipital cortex during correct > incorrect retrieval was negatively associated with working memory capacity (Fig. 4b, Table 2). Conversely, in the MPH group, increased activation in the right postcentral gyrus during correct > incorrect retrieval was positively associated with working memory capacity (Fig. 4c, Table 2).

**Table 2:** Activation and connectivity during memory retrieval 72 h after drug intake depends on working memory capacity (WMC)

		MNI			
Contrasts and brain regions	*x*	*y*	*z*	*Z* value	Cluster size
All subjects, *N* = 53 Correct > incorrect, MPH > placebo, × WMC					
L lateral occipital cortex	−52	−66	−4	4.23	739
R middle occipital gyrus	28	−80	0	4.14	177
L superior parietal gyrus	−32	−63	56	4	117
R postcentral gyrus	66	−10	18	3.78	131
R inferior occipital gyrus	35	−66	−18	3.17	133
Placebo subjects, *N* = 27 Correct > incorrect, × WMC ^*N*^					
L lateral occipital cortex	−52	−63	0	3.68	125
L fusiform gyrus	−32	−60	−18	3.58	218
MPH subjects, *N* = 26 Correct > incorrect, × WMC ^*P*^					
R postcentral gyrus	66	−14	18	3.44	179
Placebo subjects, *N* = 27 PPI, Correct > incorrect, × WMC ^*P*^					
R middle temporal gyrus	60	10	−24	3.92	153
R superior temporal gyrus	49	14	−21	3.38	
R hippocampus	28	−10	−24	3.34	
R middle temporal gyrus	66	0	−21	3.24	
R hippocampus	18	0	−24	3.14	

MNI coordinates represent the location of peak voxels. We report the first local maximum within each cluster. For the connectivity result (PPI, lower part), we report the first five local maxima within the cluster. Effects were tested for significance using cluster inference with a cluster-defining threshold of *P* < 0.005 and a cluster probability of *P* < 0.05 FWE-corrected for multiple comparisons (critical cluster sizes: all subjects, 93 voxels; placebo subjects, 89 voxels; methylphenidate (MPH) subjects, 108 voxels; placebo subjects, PPI, 110 voxels). Results from the upper analysis (all subjects, *N* = 53, correct > incorrect, MPH > placebo, × WMC) remain widely consistent using a cluster-defining threshold of *P* < 0.001: L middle temporal gyrus, *x* = −52, *y* = −66, *z* = −4, *Z* value = 4.23, cluster size: 64 voxels; L middle occipital gyrus, *x* = −28, *y* = −91, *z* = 21, *Z* value = 4.06, cluster size: 70 voxels; L inferior occipital gyrus, *x* = −21, *y* = −77, *z* = −7, *Z* value = 3.91, cluster size: 85 voxels. Results from the remaining analyses are not significant at a cluster-defining threshold of *P* < 0.001. The PPI was based on a spherical seed region in the lateral occipital cortex (*x* = −52, *y* = −63, *z* = 0; see also Materials and methods, fMRI connectivity analysis). *N* denotes a negative, and *P* a positive association between outcome and working memory capacity. Anatomical nomenclature was obtained from the Laboratory for Neuro Imaging (LONI) Brain Atlas (LPBA40; http://www.loni.usc.edu/atlases/)

*L* left, *R* right

**Fig. 5:**
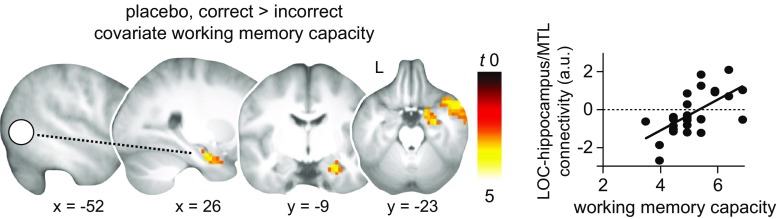
Connectivity during memory retrieval 72 h after drug intake depends on working memory capacity. Placebo subjects (*N* = 27) show increased connectivity (PPI) during correct > incorrect retrieval between the left lateral occipital cortex (LOC; 8 mm sphere around peak coordinate: *x* = −52, *y* = −63, *z* = 0; marked as *filled white circle*; Materials and methods, fMRI connectivity analysis) and the right hippocampus, including surrounding medial temporal (MTL) structures, at higher working memory capacity. Connectivity is schematically illustrated through the *dashed line*. For visualization purposes, the scatter plot shows the relationship between working memory capacity and LOC-hippocampal/MTL connectivity during correct > incorrect retrieval (% signal change, arbitrary units, a.u.), extracted from the significant cluster. Results are shown at *P* < 0.005 (*P* < 0.05, FWE-corrected at cluster level; see also Table 2). Sliced images are based on the average structural scan of all subjects. *L* left

In addition to levels of activation, drug administration might differentially affect retrieval-related connectivity depending on individual working memory capacity. Thus, we performed connectivity analyses (PPI) and placed seeds in the left lateral occipital cortex for placebo subjects and in the right postcentral gyrus for MPH subjects (see above, and Materials and methods, fMRI connectivity analysis). Results revealed increased functional coupling between the left lateral occipital cortex and the right hippocampus (and surrounding medial temporal lobe structures) during correct compared to incorrect retrieval to be positively associated with working memory capacity in the placebo group (Fig. 5, Table 2). There was no negative relationship with working memory capacity. Also, there was no significant association between connectivity profiles of the right postcentral gyrus and working memory capacity in the MPH group.

In sum, the relationship of working memory capacity with activation and connectivity during correct retrieval differed between the groups. In the placebo group, correct memory retrieval was associated with increased activation in the left lateral occipital cortex and fusiform gyrus, as well as decreased connectivity between the left occipital cortex and the hippocampus at *lower* working memory capacity. In the MPH group, correct memory retrieval was associated with increased activation in the postcentral gyrus at *higher* working memory capacity.

## Discussion

The present study investigated the effects of catecholaminergic modulation on the synaptic and subsequent systems consolidation of associative memories in humans. Contrary to what we expected, MPH administration after learning did not facilitate long-term associative memory compared to placebo. Also, there was no significant group difference in activation during delayed memory retrieval after 72 h. However, we found that the effect of drug administration on subsequent retrieval-related activation and connectivity was dependent on individual variations in working memory capacity.

We expected that the up-regulation of catecholaminergic signaling during early consolidation would stabilize initially fragile memories that would otherwise decay and thus lead to enhanced long-term associative memory performance following MPH compared to placebo administration. This would provide evidence for a synaptic tagging and capture mechanism in humans. Contrary to our expectations, we did not find increased associative memory performance for the MPH relative to the placebo group after 72 h (Fig. 2). There are several possible explanations for this null finding. First, MPH administration might have been efficient but was masked by other experimental factors. For instance, receiving a pill, even if it only was a placebo, could have been sufficiently arousing to facilitate the release of endogenous catecholamines and to foster synaptic consolidation. Second, the dosage of 20 mg MPH might not have been sufficient to facilitate catecholaminergic signaling in order to augment synaptic consolidation after learning. Although we chose the absolute dose of 20 mg MPH in accordance with previous studies (Izquierdo et al. 2008; Linssen et al. 2012), the average relative body-weight-adjusted dose of 0.27 mg/kg MPH was rather low, compared to the dose typically used to investigate the effects of MPH on brain function (∼0.8 mg/kg; Volkow et al. 2001). Nevertheless, MPH administration affected heart rate (Fig. 2) but did not significantly affect blood pressure or mood (see also Volkow et al. 2002). Third, the timing of drug administration was perhaps not optimal to facilitate synaptic consolidation, and catecholamine levels could have peaked earlier (e.g., after the first study cycle) or later (Izquierdo et al. 2008). Further research is needed to clarify these points.

Next, we hypothesized that synaptic consolidation would pave the way for the subsequent neocortical stabilization of memory traces (Frey and Morris 1997; Frankland and Bontempi 2005; Lesburguères et al. 2011). As expected, results revealed stronger activation in the medial prefrontal and posterior cingulate cortex, the angular gyrus, and posterior representational regions but also in the hippocampus, during correct relative to incorrect memory retrieval after 72 h (Fig. 3). However, we did not find increased engagement of neocortical regions in subjects who received MPH compared to placebo. Notably, there was no disengagement of the hippocampus during the delayed retrieval which is probably due to the spatial associative nature of our task (Burgess et al. 2001; Düzel et al. 2003; Mayes et al. 2007).

Although catecholamine administration during early consolidation did not foster long-term associative memory performance, we found that the effects of MPH on retrieval-related neuronal processes were dependent on individual variations in working memory capacity, a proxy measure for baseline catecholamine synthesis capacity (Cools et al. 2008; Landau et al. 2009). Subjects who received placebo showed increased activation in the left lateral occipital and fusiform regions but decreased connectivity between the left lateral occipital cortex and the right hippocampus at *lower* working memory capacity during correct over incorrect retrieval (Figs. 4 and 5). Conversely, for subjects who received MPH, increased activation in the right postcentral gyrus was associated with *higher* working memory capacity (Fig. 4). Behaviorally, we did not find an interaction between working memory capacity and associative memory performance.

Prior studies have shown that inter-individual differences in drug response can be explained by working memory capacity (Kimberg et al. 1997; Mehta et al. 2000; van der Schaaf et al. 2013). While cognitive performance in subjects with low working memory capacity typically benefits from administration of catecholaminergic stimulants (Mehta et al. 2000; Gibbs and D’Esposito 2005), they can have detrimental effects in subjects with high working memory capacity (Kimberg et al. 1997). This speaks for an inverted u-shaped relationship (Williams and Goldman-Rakic 1995; Cools and D’Esposito 2011). Here, we suggest that subjects with low working memory capacity fall on the left-hand side of the inverted u-shaped curve, and subjects that score high are located in the middle (i.e., optimal baseline catecholamine synthesis capacity). Although higher working memory capacity was not associated with better long-term memory performance, it was related to enhanced connectivity between the left lateral occipital cortex and the hippocampus but decreased activation in lateral occipital cortex and fusiform gyrus during correct memory retrieval in subjects who received placebo. Presumably, increased cross-talk reflects better integration of mnemonic features into a conjunctive, episodic-like memory representation and memory stabilization within an extended hippocampal-neocortical network. This might indicate more efficient neuronal processing (Gibbs and D’Esposito 2005; Rypma et al. 2006). Therefore, subjects with high working memory capacity might already display optimal grounds for synaptic and subsequent systems consolidation. Furthermore, an additional dose of MPH should be detrimental rather than beneficial for neuronal processing in subjects with already optimal baseline catecholamine levels. Indeed, for subjects who received MPH, higher working memory capacity was associated with increased activation in the right postcentral gyrus. Our results thus imply that MPH administration affected the consolidation of long-term memory traces depending on baseline catecholamine levels. This lead to differential retrieval processes for correct, associative memories after 72 h. One possible limitation is that our between-subject design might have reduced the sensitivity of our baseline-dependent analysis. A follow-up study may employ a within-subject manipulation to disentangle the effects of MPH from subject-specific variance.

Overall, we did not find evidence for increased long-term associative memory performance in MPH compared to placebo subjects, and groups did not show differential activation during memory retrieval after 72 h. However, these results do not preclude the presence of a synaptic tagging and capture mechanism in humans. Most importantly, we found that MPH administration after learning affected long-term retrieval-related neural processes depending on individual differences in catecholamine synthesis capacity at baseline.
